# Effectiveness of a community-based positive prevention intervention for people living with HIV who are not receiving antiretroviral treatment: a prospective cohort study

**DOI:** 10.9745/GHSP-D-12-00023

**Published:** 2013-03-21

**Authors:** Avina Sarna, Stanley Luchters, Eustasius Musenge, Jerry Okal, Matthew Chersich, Waimar Tun, Sabine Mall, Nzioki Kingola, Sam Kalibala

**Affiliations:** a HIV and AIDS Program, Population Council, Delhi, India; b International Centre for Reproductive Health, University of Ghent, Belgium; c Faculty of Health Sciences, University of Witwatersrand, Johannesburg, South Africa; d Burnet Institute, Melbourne, Victoria, Australia; e Monash University, Victoria, Australia; f HIV and AIDS Program, Population Council, Nairobi, Kenya; g HIV and AIDS Program, Population Council, Washington, DC, USA; h International Centre for Reproductive Health, Mombasa, Kenya

## Abstract

**Background:** We report effectiveness of an HIV-prevention intervention delivered by community health workers (CHWs) in Mombasa, Kenya, to PLHIV who have not initiated or who have discontinued ART—an often difficult-to-reach population because they fall outside the ambit of health care and prevention services.

**Methods:** A 2-arm cohort study assessed a structured risk-reduction intervention involving at least 4 one-to-one counseling sessions and personalized support. The control group received standard prevention services. CHWs recruited treatment-naïve people living with HIV (PLHIV) or those who had previously taken antiretroviral drugs. Data were analyzed using a Propensity Score Matched (PSM)-sample to control for baseline differences between the groups.

**Results:** 634 PLHIV were recruited and followed for 6 months. Median age was 35 years, and 74.3% were female. Participants in the intervention group reported reduced risky sexual behaviors both at endline compared with baseline and compared with the control group. At endline, in the PSM analysis, participants in the intervention arm were less likely than participants in the control group to report unprotected sex with a spouse (Odds Ratio [OR] = 0.08, 95% confidence interval [CI] = 0.03-0.24), and they reported fewer unprotected sex acts (12.3% versus 46.0%, respectively; OR = 0.16, 95% CI = 0.09-0.29; *P*<0.001). Further, 92.4% of participants in the intervention group reported zero unsafe sex acts (with partners of negative or unknown HIV status) compared with 70.8% in the control group (*P*<0.001), and more participants in the intervention arm were receiving ART (34.3% versus 12.7%, respectively; *P*<0.001).

**Conclusion:** CHWs effectively reached PLHIV who had never received or who had discontinued ART, and they delivered a risk-reduction intervention that led to declines in reported sexual risk behaviors, as well as to increases in ART uptake. A scaled-up intervention warrants consideration.

## Abstract (precis)

In Mombasa, Kenya, a community-based HIV risk-reduction intervention effectively reached people living with HIV who were not receiving antiretroviral treatment (ART)—a difficult-to-reach population because they often fall outside the ambit of health care services—and succeeded in reducing reported risky sex behavior and increasing ART uptake.

## INTRODUCTION

People living with HIV (PLHIV) comprise those who do and those who do not know their HIV status; both groups constitute important populations for HIV prevention. Among those who know their status, PLHIV who receive antiretroviral therapy (ART) and those who are not yet eligible for treatment but access HIV care for regular follow-up, such as for co-trimoxazole prophylaxis, have frequent contact with health services and are exposed to prevention information and commodities. However, many PLHIV have infrequent or no contact with health services, mostly because they are not yet eligible for treatment, have not had contact with an ART center, or have declined or discontinued ART.

Many PLHIV have infrequent or no contact with health services.

HIV-prevention programs in low- and middle-income countries rarely have direct contact with PLHIV not accessing services, apart from mass media campaigns, for example. There are very few positive prevention studies (prevention aimed at PLHIV) among PLHIV who know their status but are not accessing treatment. Our previous study showed that high-risk sexual behaviors are common in this group, which can place PLHIV at higher risk of superinfection or of acquiring new sexually transmitted infections (STIs) and put their partners without HIV at risk of infection. Unprotected sex occurred in about half of sexual partnerships, notably one-third of them with partners without HIV and slightly more than one-half with partners of unknown HIV status.^1^

To date, most HIV-prevention studies targeting populations with HIV in the United States^2-4^ and developing countries^5-9^ have been among those accessing health services, such as HIV testing and counseling (HTC) centers, HIV care or treatment services, family planning clinics, maternal and child health clinics, or STI services. Interventions evaluated in these studies varied in frequency, delivery format, and content, and covered a combination of risk assessment, risk reduction, partner testing, HIV-status disclosure, and promotion of condom use. A systematic review and meta-analysis of HIV-prevention interventions suggest that interventions tend to be more successful in reducing risky behaviors if they are based on behavioral theory, specifically designed to change HIV-transmission risk behaviors, and delivered to individuals in an intensive manner and with skills building by health care providers in service settings.^4^

Positive prevention interventions focus on reducing risky sex behaviors among PLHIV.

In 2010, in low- and middle-income countries, ART coverage was around 47% among the 14.2 million eligible persons.^10^ In Kenya, in 2009, of the estimated 1.4 million PLHIV, 438,000 had advanced disease (defined as CD4 <200 cells/mm^3^) and were eligible for treatment. About 309,000 were receiving ART.^11^ These figures show that the majority of PLHIV, many of whom are not yet eligible for ART, fall outside the ambit of regular health care and preventive services. Many PLHIV do not reach or access care services after their HIV tests, with early pre-ART losses of up to 33% among newly diagnosed PLHIV.^12-14^

An estimated 100,000 new adult HIV infections occurred in 2009 in Kenya,^11^ highlighting the need for intensive combination prevention efforts, including those focused on the sexual risk behaviors of PLHIV, the large majority of whom do not access HIV care services. In this paper, we present findings from a 2-arm cohort study in Mombasa, Kenya, with pre- and post-measures. This controlled study aimed to assess the effectiveness of a personalized HIV risk-reduction intervention delivered by community health workers (CHWs) to PLHIV who know they have HIV and who are not on treatment. The intervention was aimed primarily at reducing the number of unprotected sex acts and sexual partners and to increase disclosure. Secondarily, we assessed the effects of the intervention on stigma and ART uptake. This paper addresses a key population that has not been studied and adds important evidence to current literature on combination Prevention with Positives (PwP).

## METHODS

### Samples and Procedures

The study was conducted in Changamwe and Likoni Divisions of Mombasa, Kenya. The 2 divisions are geographically distinct, approximately 11 km apart, but they have a similar HIV prevalence (about 6% of adults in a 2010 sentinel survey^15^). The divisions also have comparable commercial activity related to the port, tourism industry, and small-scale businesses, and they have similar networks of health centers, HTC centers, and CHWs.

CHWs have a wide reach in the community through various programs (for example, antenatal care, social support, and nutrition services from faith-based organizations) and could theoretically reach PLHIV assigned to both the control and treatment groups. Thus, to retain intervention integrity and avoid contamination, participants recruited from Changamwe Division were assigned to the intervention arm and those recruited from Likoni, to the control arm. Participants in both groups were followed for 6 months. Baseline and endline data were collected in both groups.

Study participants were recruited by CHWs using non-probability targeted sampling, an approach where field-based outreach workers actively recruit participants from identified geographic areas and populations of interest based on a prior ethnographic assessment.^16^ CHWs are lay health workers used widely in community-based programs, including social support services, and are familiar with the community and its socio-demographic profile. Local CHWs were asked to undertake a descriptive ethnographic assessment of the population in the selected study areas. Information was sought on socio-economic status, tribes, religious groups, and presence of high-risk groups and PLHIV.

PLHIV in Mombasa are poorly networked and reluctant to reveal their status.^17^ As there is no listing of PLHIV in the community, each CHW first identified 2 to 3 unrelated index PLHIV and then asked the index PLHIVs to connect the CHW to other PLHIV they knew. The CHW identified a new index PLHIV if the previous PLHIV did not know any other PLHIV. To limit biases related to recruiter characteristics and consequent oversampling of participants with similar characteristics, each CHW could recruit up to 20 participants. This allowed all CHWs in the study to recruit PLHIV from their communities and networks while avoiding the potential for some CHWs to recruit a large and disproportionate number of PLHIV from a single group that they served (such as female sex workers, men who have sex with men, or injection drug users [IDUs]).

Ethical approval was obtained from the Kenyatta National Hospital's Ethics Committee in Kenya and the Institutional Review Board of the Population Council in the United States. Recruitment followed a detailed protocol on approaching PLHIV, maintaining confidentiality, and verifying the participant's HIV status by checking HIV/CD4 test results or the referral card issued by an HCT center.

CHWs approached PLHIV in the community they served and arranged a time and convenient place to meet. CHWs then confirmed their HIV status, provided information on the study, and assessed willingness to participate and be followed over 6 months. Following this, the CHW fixed a time to accompany the potential participant to the study site. Trained study staff then assessed participants for eligibility and enrolled participants after completing informed consent procedures. Eligible participants included residents of Changamwe or Likoni who were sexually active (had sex at least once in the past 3 months) adults with HIV and were either ART naïve or had taken antiretroviral drugs at least 6 months previously for any indication, including ART or prevention of mother-to-child transmission of HIV (PMTCT).

### Intervention Description

CHWs in the intervention arm underwent intensive 7-day training on study procedures and the national orientation package for community HIV service providers.^18^ These CHWs were trained in counseling methods and motivational techniques to assist clients with HIV to identify barriers to safe sex that they faced, to help clients discuss strategies to overcome these barriers and set goals until the next meeting, and to encourage and support clients in achieving these goals. CHWs from the control site were trained for 3 days on study procedures and PLHIV recruitment. They also received training on the intervention after study completion.

The intervention, provided over a period of 6 months, consisted of a minimum of 4 structured CHW meetings with the participant, each meeting lasting 30–60 minutes. No maximum number of visits was stipulated. In these sessions, CHWs counseled participants on HIV/STI risk reduction and treatment, consistent and correct use of condoms, disclosure of HIV status to partners, HIV testing for partners and children, registering for HIV treatment, family planning, and PMTCT.

The participants and CHWs met at a mutually convenient location, for instance, at a health center, HTC center, post-test club, or the participant's home. CHWs used flipcharts to maintain uniformity of message content and to demonstrate correct usage of condoms. All counseling and contact with participants was conducted in individual one-to-one sessions. The spouse or main sexual partner could participate during pre-arranged couple counseling sessions with the consent of the study participant. See box for details of the counseling schedule and content.

**Box. Description of Planned Activities During Each Counseling Visit**

**Visit 1:**

- Personalized assessment of risk behaviors
- Identification of specific areas of need, for example, condom-related misconceptions, non-disclosure of HIV status to spouse/main partner
- Overview of HIV transmission and prevention strategies using a flipchart

**Visit 2:**

- Review of key prevention needs
- Motivation on risk reduction and goal setting
- Assistance with counseling for untested partners and facilitation of HIV testing for partners
- Facilitation of referrals to services for prevention of mother-to-child transmission of HIV (PMTCT), family planning, or treatment of sexually transmitted infections (STIs) per need
- Counseling on need for registration at HIV treatment and care center

**Visit 3:**

- Review of progress toward goals set during previous visit
- Follow-up on referrals made to STI or family planning clinics, HIV treatment and care centers, or PMTCT services
- Discussion of and referrals for testing of children and other family members

**Visit 4:**

- Review of key prevention message
- Follow up on disclosure, risk-reduction goals, and partner testing

Participants could request additional visits.

CHWs contacted participants in the control arm at baseline and endline. These CHWs continued to provide routine support services to PLHIV in the control group but without a specified visit schedule or defined counseling content. Data on the number of CHW encounters with controls were not collected.

CHWs were paid a stipend of KSh2,000 (US$27) per month based on the national guidelines, plus reimbursement for transportation costs. Study participants received KSh150 (US$2) as compensation for time spent completing the baseline and endline assessments.

### Data Collection

Participants in both groups completed baseline (pre-intervention) and endline (post-intervention) assessments. Data were collected on socio-demographic characteristics, sexual behavior, perceived stigma, and receipt of ART through face-to-face interviews, conducted in Swahili by trained interviewers. Data were entered using Computer-Assisted Personal Interview (CAPI). However, information on more sensitive behaviors was collected by the participants themselves using Audio Computer-Assisted Self-Interview (ACASI); the interviewer left the room after guiding the participant on the technique.^19^ All response options were color-coded and linked to audio directions in Swahili. The interviewer was available outside the door to help the participant in case s/he was unable to understand a particular question or had difficulty with the computer program.

### Measures

Sexual behavior data were collected on the number and type of sexual partners, condom use, and disclosure of own status to partners. Participants could report on up to 6 of their most recent sexual partners in the 3 months prior to the survey. A regular partner was defined as a cohabiting partner, and a casual partner as a partner with whom the participant was not living and had sex with once or rarely. A commercial partner was one in which money or gifts were exchanged for sex. Concurrent sexual relationships were based on overlapping dates for 2 or more partners.

While unprotected sex is considered an important outcome, with unprotected sex between 2 PLHIV carrying risks, the study focused on the outcome of unsafe sex. This outcome, a subset of unprotected sex, was defined as sex without condoms with a partner who is of negative or unknown/untested HIV status.

Condom use fatigue was assessed using a 4-item Likert scale from strongly agree to strongly disagree with the statement: “I am tired of always having to make sure that I use a condom every time I have sex.”^1^ Condom use self-efficacy was assessed using a 15-item scale (Cronbach's alpha = 0.79) derived from the Condom Use Self-Efficacy Scale (CUSES) (Cronbach's alpha = 0.91).^20-21^ The scale included items assessing the mechanics of using a condom, partner's disapproval of using a condom, assertiveness, and condom use under the influence of intoxicants. Participants responded on a 5-item Likert scale from strongly agree to strongly disagree. Total scores (possible range 15–75) were categorized as low (15–34), moderate (35–54), or high self-efficacy (55–75).

Perceived internalized stigma was assessed using a 16-item scale (Cronbach's alpha of adapted scale = 0.81) derived from Berger's HIV stigma scale (Cronbach's alpha = 0.96).^22^ The scale covered 4 domains with 4 items for each domain: personalized stigma, disclosure concerns, negative self-image, and public attitudes. Participants responded on a 4-item Likert scale from strongly agree to strongly disagree. Total scores (possible range 16–64) were categorized as low (16–40), moderate (41–52), or high stigma (53–64).

A 5-item test with a composite total correct response score was used to assess HIV knowledge. The test included items on transmission of HIV through mosquito bites, transmission through sharing of utensils, transmission from mother to child, reduction of HIV-transmission risk with ART, and re-infection with new viral strains. Concerns about HIV transmission were assessed using 2 statements (4-item Likert scale from strongly agree to strongly disagree) derived from an HIV treatment optimism-skepticism scale: “New treatments take the worry out of sex” and “I am less concerned about infecting sexual partners.”^23^

### Data Analysis

Data were analyzed at 2 levels: within each group to document changes in key sexual and behavioral outcomes over time and between groups at endline. Stata 12.1 was used for data analysis.

Preliminary analyses showed that there were imbalances between some socio-demographic variables in the intervention and control groups, which potentially introduce selection bias. We addressed this by using propensity score matching (PSM), a technique useful for estimating causal effects in non-randomized studies and for removing selection bias.^24^^,^^25^

To select variables to include in matching, we used a multivariate logistic regression model to calculate the participants' propensity to being included in the intervention group. The intervention or control group was thus the model outcome, and explanatory variables were the variables that were unbalanced or likely to be associated with exposure to the study intervention.

Post-logistic regression we extracted the propensity scores, which showed different distributions in the 2 arms. Finally, Kernel caliper matching was conducted on 5 variables: age, gender, education, religion, and employment.^26^ Matching was effective in balancing all variables. Statistical analyses were conducted on the original and matched data.

Pearson's chi-square test, *t*-test, and Mann-Whitney tests were used to compare groups on categorical and continuous variables at baseline. Wilcoxon signed rank tests, McNemar test, and tests of marginal homogeneity (Stuart-Maxwell and linear trend) for repeated measures on 2 related samples were used to document change within groups over the 2 time periods. Pearson's chi-square test, chi-square test for trend, and Wilcoxon rank sum test were used to compare outcomes between the intervention and control groups 6 months after the intervention. Finally, we computed unadjusted odds ratios and 95% confidence intervals (CI), comparing main study outcomes between intervention and control groups for the PSM samples. For all tests performed, *P* values <0.05 were considered statistically significant.

## RESULTS

### Participant Recruitment and Retention

**FIGURE 1.**
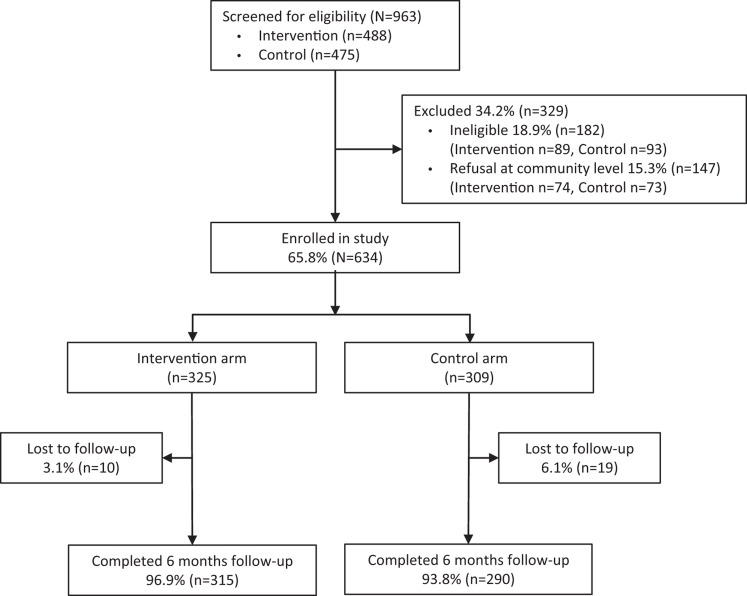
Flow Diagram of Progress of HIV-Infected Study Participants Through the Study

At recruitment, 147 persons refused to participate in the study, 15.2% in the intervention group and 15.4% in the control site; information on the gender or socio-demographic profile of these persons is not available. A total of 634 participants were enrolled (February–May 2010) and 605 (95.4%) completed 6 months of follow-up, forming the analytic sample for the effectiveness analysis (Figure 1). In the intervention group, 178 (56.5%) of the participants who completed the study received the minimum 4 contact visits from CHWs, a further 136 participants (43.2%) were visited 5 times, and 1 participant was visited 6 times.

Table 1 provides details of the baseline characteristics of enrolled participants and participants in the PSM sample (N = 394). The average age of study participants in each group was 35 years, and 76.9% of participants in the intervention group and 71.5% in the control group were women (*P* = 0.12). Almost two-thirds of the participants had only primary education while 28% had a secondary or higher education (*P* = 0.87 comparing education distribution in the 2 groups).

**Table 1.** Baseline Characteristics of HIV-Infected Adults Enrolled in Intervention and Control Sites in Mombasa, Kenya, and of a Propensity Score-Matched Selected Population

Variable	Enrolled Population (N = 634)			Propensity Score-Matched Population (N = 394)^a^		
	Intervention (n = 325)	Control (n = 309)	*P* Value^b^	Intervention (n = 204)	Control (n = 190)	*P* Value^b^
**Socio-Demographic Characteristics**						
Female, n (%)	250 (76.9)	221 (71.5)	0.12	157 (77.0)	150 (79.0)	0.64
Age, mean years (SD)	35.2 (8.4)	35.6 (8.2)	0.51^c^	35.1 (8.6)	34.6 (7.9)	0.54^c^
Education, n (%)						
No schooling	30 (9.2)	30 (9.7)		18 (8.8)	21 (11.1)	
Primary education	206 (63.4)	190 (61.5)		130 (63.7)	109 (57.4)	
Secondary or higher education	89 (27.4)	89 (28.8)	0.87	56 (27.4)	60 (31.6)	0.42
Marital status, n (%)						
Married or cohabiting	98 (30.2)	165 (53.4)		67 (32.8)	96 (50.5)	
Single	78 (24.0)	47 (15.2)		48 (23.5)	29 (15.4)	
Separated or divorced	80 (24.6)	51 (16.5)		45 (22.1)	35 (18.4)	
Widowed	69 (21.2)	46 (14.9)	<0.001*	44 (21.6)	30 (15.8)	0.004*
Living arrangements, n (%)						
Stays alone	163 (50.2)	67 (21.7)		94 (46.1)	47 (24.7)	
Nuclear family	103 (31.7)	193 (62.5)		70 (34.3)	116 (61.1)	
Extended family/friends	59 (18.2)	49 (15.9)	<0.001*	40 (19.6)	27 (14.2)	<0.001*
Religion, n (%)						
Catholic	120 (36.9)	35 (11.3)		49 (24.0)	31 (16.3)	
Protestant	135 (41.5)	109 (35.3)		93 (45.9)	101 (53.2)	
Muslim	66 (20.3)	164 (53.1)		62 (30.4)	57 (30.0)	
No religion	4 (1.2)	1 (0.3)	<0.001*	…	1 (0.5)	0.17
Employment, n (%)						
Salaried job or self-employed	56 (17.2)	33 (10.7)		29 (14.2)	27 (14.2)	
Daily wage worker	88 (27.1)	71 (23.0)		61 (29.9)	50 (26.3)	
Vendor or hawker	55 (17.0)	76 (24.5)		50 (24.5)	51 (26.8)	
Green grocer	15 (4.6)	10 (3.2)		7 (3.4)	7 (3.7)	
Unemployed	49 (15.1)	49 (15.9)		36 (17.7)	34 (17.9)	
Other	34 (10.5)	36 (11.7)	<0.001*	21 (10.3)	21 (11.1)	0.98
**HIV-Related Characteristics**						
Number HIV tests done, n (%)^e^						
1	165 (50.8)	207 (67.2)		104 (51.0)	126 (66.3)	
2-4	139 (42.8)	92 (29.9)		87 (42.7)	58 (30.5)	
5 or more	21 (6.5)	9 (2.9)	<0.001*	13 (6.4)	6 (3.2)	0.007*
Months since HIV-positive diagnosis, n (%)^e^						
0-11	83 (25.9)	102 (34.6)		51 (25.4)	61 (34.1)	
12-23	74 (23.1)	69 (23.4)		46 (22.9)	42 (23.5)	
24 or more	163 (50.9)	124 (42.0)	0.040*	104 (51.7)	76 (42.5)	0.12
Ever taken antiretroviral drugs (including for PMTCT), n (%)	125 (38.5)	119 (38.5)	0.59	75 (36.8)	66 (34.7)	0.68
Currently attends HIV clinic	4 (1.2)	14 (4.5)	0.012*	3 (1.5)	9 (4.7)	0.06
Disclosed HIV status to main partner, n (%)^e^						
Yes	160 (51.3)	203 (70.7)		102 (52.0)	121 (68.8)	
No, but plans to disclose	67 (21.5)	41 (14.3)		39 (19.9)	29 (16.5)	
No, does not intend to disclose	46 (14.7)	27 (9.4)		30 (15.3)	17 (9.7)	
No, cannot say/maybe will disclose	39 (12.5)	16 (5.6)	<0.001*	25 (12.8)	9 (5.1)	0.004*
Disclosed HIV status to anyone besides health workers, n (%)	245 (75.4)	272 (88.0)	<0.001*	157 (77.0)	164 (86.3)	0.02*
**Sexual Behavior Characteristics**						
Age at first sex, median n (IQR, range)	18 (15–19, 7–42)	18 (15–19, 7–35)	0.91^d^	18 (9–25, 7–42)	17 (11–26, 7–35)	0.30^d^
Number lifetime partners, median n (IQR, range)	5 (3–9, 1–53)	4 (3–9, 1–57)	0.49^d^	5 (1–20, 1–30)	4 (1–35, 1–57)	0.28^d^
Ever had same sex partners, n (%)	17 (5.2)	15 (4.9)	0.86	8 (3.9)	10 (5.3)	0.52
Has regular sexual partner(s), n (%)	312 (96.0)	287 (92.7)	0.093	79 (42.9)	44 (24.9)	<0.001*
Has children, n (%)	281 (86.5)	265 (85.8)	0.80	176 (86.3)	162 (85.3)	0.77
Currently using family planning, n (%)	187 (57.5)	163 (52.8)	0.46	112 (58.0)	96 (53.9)	0.33

Abbreviations: SD, standard deviation; PMTCT, prevention of mother-to-child transmission; IQR, interquartile range.

* *P* values < 0.05 were considered statistically significant.

a The following variables were used in propensity score matching: age, gender, education, religion, and employment.

b Pearson's chi-square test unless otherwise indicated.

c *t*-test.

d Mann-Whitney test.

e For the enrolled population, sample size of control group for number of HIV tests done is 308; of intervention and control group for months since HIV-positive diagnosis is 320 and 295, respectively; of intervention and control group for disclosed HIV status to main partner is 312 and 287, respectively.

At baseline, differences were detected between the intervention and control groups on a number of characteristics. Notably, at baseline, participants in the intervention group were more likely than those in the control group to be single or separated, to live alone, to be Catholic/Protestant, and to report multiple HIV tests. They were less likely to disclose their HIV status to partners or others (Table 1).

In both groups, 38.5% of participants had taken antiretroviral drugs previously (either for ART or PMTCT) and a small number attended an HIV clinic. Only a single participant reported injection drug use in the past 6 months (data not shown). The majority of participants in both groups had regular sexual partners (>92%; *P* = 0.093) and biological children (86.5% in intervention and 85.8% in controls; *P* = 0.80), and more than half of participants in each group were currently using family planning.

### Behavior Change in Study Sample

Compared with baseline, at endline, the proportion of participants in the intervention group reporting a number of **risky sexual behaviors** declined considerably, including having multiple partners (2 or more partners in the past 3 months), concurrent relationships in the past 3 months (41.7% at baseline versus 18.2% at endline; *P* = 0.015; data not shown), unprotected sex with various partners at last sex, unprotected sex acts in the last month, and unsafe sex in the last month (with partners of negative or unknown HIV status) (Table 2).

**Table 2.** Sexual and Behavioral Outcomes Among HIV-Infected Men and Women Before and 6 Months After a Behavioral Intervention in Mombasa, Kenya (N = 605)

Variable	Intervention (n = 315)			Control (n = 290)		
	Before n/N (%)	After n/N (%)	*P* Value^a^	Before n/N (%)	After n/N (%)	*P* Value^a^
Number of partners in past 3 months						
0 partners	11/297 (3.7)	58/313 (18.5)		9/283 (3.2)	54/286 (18.9)	
1 partner	158/297 (53.2)	202/313 (64.5)		201/283 (71.0)	174/286 (60.8)	
≥2 partners	128/297 (43.1)	53/313 (16.9)	<0.001*^b^	73/283 (25.8)	58/286 (20.3)	0.06^b^
Unprotected sex at last sexc,d						
Spouse	68/93 (73.1)	9/79 (11.4)	<0.001*	97/144 (67.4)	85/141 (60.3)	0.19
Regular partner	71/115 (61.7)	4/92 (4.4)	<0.001*	56/125 (44.8)	54/105 (51.4)	1.00
Casual partner	89/142 (62.7)	11/101 (10.9)	<0.001*	15/31 (48.4)	20/33 (60.6)	1.00
Commercial partner	36/53 (67.9)	2/33 (6.1)	0.025*	25/43 (58.1)	13/28 (46.4)	0.025*
Unprotected sex in past monthd,e						
0 acts	115/307 (37.5)	225/258 (87.2)		115/282 (40.8)	126/238 (52.9)	
1-5 acts	87/307 (28.4)	28/258 (10.9)		102/282 (36.1)	69/238 (29.0)	
≥6 or more acts	105/307 (34.2)	5/258 (1.9)	<0.001*^b^	65/282 (23.1)	43/238 (18.1)	0.002*^b^
Unsafe sex in past monthd,e						
0 HIV-negative or unknown status partner	137/307 (44.6)	240/258 (93.0)		172/282 (61.0)	168/238 (70.6)	
1 HIV-negative or unknown status partner	23/307 (7.5)	4/258 (1.6)		26/282 (9.2)	10/238 (4.2)	
2-5 HIV-negative or unknown status partners	59/307 (19.2)	10/258 (3.9)		43/282 (15.3)	33/238 (13.9)	
≥6 HIV-negative or unknown status partners	88/307 (28.7)	4/258 (1.6)	<0.001*^b^	41/282 (14.5)	27/238 (11.3)	0.003*^b^
Condom use self-efficacy						
Low self-efficacy (score 15-34)	23/315 (7.3)	2/315 (0.6)		8/290 (2.8)	0/290 (0)	
Moderate self-efficacy (score 35-54)	169/315 (53.7)	31/315 (9.8)		129/290 (44.5)	147/290 (50.7)	
High self-efficacy (score 55-75)	123/315 (39.1)	282/315 (89.5)	<0.001*	153/290 (52.8)	143/290 (49.3)	0.86
HIV status disclosed to partnerc,d						
Spouse	65/93 (70.0)	74/79 (93.7)	<0.001*	120/144 (83.3)	125/141 (88.7)	0.083
Regular partner(s)	16/115 (13.9)	8/92 (8.7)	0.65	11/125 (8.8)	16/105 (15.2)	0.41
Casual partner(s)	19/142 (13.4)	10/101 (9.9)	1.00	7/31 (22.6)	12/33 (36.4)	1.00
Commercial partner(s)	8/53 (15.1)	7/33 (21.2)	0.16	6/43 (14.0)	7/28 (25.0)	0.56
Currently receiving antiretroviral treatment	1/315 (0.3)	111/315 (35.2)	<0.001*	1/290 (0.3)	36/290 (12.4)	<0.001*
Internalized stigma						
Low (score 16-40)	78/314 (24.8)	133/314 (42.4)		152/290 (52.4)	172/290 (59.3)	
Moderate (score 41-52)	232/314 (73.9)	181/314 (57.6)		134/290 (46.2)	113/290 (39.0)	
High (score 53-64)	4/314 (1.3)	0/31 (0)	<0.001*	4/290 (1.4)	5/290 (1.7)	0.09

* *P* values < 0.05 were considered statistically significant.

a McNemar test unless otherwise indicated.

b Marginal homogeneity (Stuart-Maxwell) test.

c Multiple-response question.

d Respondents were asked to report on all sexual partners in the past 3 months with a maximum of 6 partners.

e Only among sexually active participants.

The percentage of study participants who reported risky sex behaviors declined over time.

In the control group, fewer and smaller changes were observed. However, significant reductions were observed in the percentage of control participants reporting unprotected sex at last sex with sex workers (*P* = 0.025), unprotected sex in the past month (*P* = 0.002), and unsafe sex in the last month (*P* = 0.003) (Table 2).

**Condom use** fatigue was reported by around half the participants and did not significantly change over time in either group (data not shown). In the intervention group, an increasing proportion of participants reported high condom use self-efficacy (39.1% at baseline versus 89.5% at endline; *P*<0.001) while there was no increase in the control group.

The percentage of intervention participants reporting **disclosure of HIV status** to their spouse increased significantly from baseline to endline (70.0% versus 93.7%; *P*<0.001) and to some extent in the control group (83.3% versus 88.7%; *P* = 0.083). In both groups, however, no statistically significant change was detected in disclosure to other types of partners. Internalized **stigma** scores declined in the intervention arm (*P*<0.001).

**ART uptake**, negligible in both populations at baseline, increased in the intervention (0.3% versus 35.2%; *P*<0.001) and control (0.3% versus 12.4%; *P*<0.001) groups over time, but it was almost three-fold higher in the intervention group than in the control group at endline (*P*<0.001). Overall, ART-experienced participants were more likely to initiate ART compared with ART-naïve participants (56.5% versus 32.6%; *P*<0.001).

ART uptake increased significantly over time in both intervention and control groups but more so in the intervention group.

### Behavior Change in Propensity Score Matched-Sample

Table 3 presents sexual and behavioral outcomes at endline among men and women with HIV in the intervention and control groups in the PSM sample. At endline, fewer participants in the intervention arm than in the control arm reported **unprotected sex at last sex**: with a spouse (9.1% versus 56.1%; Odds Ratio [OR] = 0.08, 95% confidence interval [CI] = 0.03-0.24), with a regular partner (5.1% versus 48.5%; OR = 0.06, 95% CI = 0.01-0.24), with a casual partner (15.4% versus 60.0%; OR = 0.12, 95% CI = 0.04-0.39), and with a sex worker (8.7% versus 47.4%; OR = 0.11, 95% CI = 0.02-0.72). Overall, participants in the intervention group reported fewer numbers of **unprotected sex acts in the past month** than participants in the control group (*P*<0.001). Also, they were less likely to report **unsafe sex** with any partner with negative or unknown HIV status in the last month (*P*<0.001); 92.4% of the intervention participants reported no unsafe sex acts compared with 70.8% of control-group participants.

**Table 3.** Propensity Score-Matched^a^ Sexual and Behavioral Outcomes Among HIV-Infected Men and Women After a 6-Month Behavioral Intervention (N = 394)

Variable	End of study		
	Intervention n/N (%)	Control n/N (%)	*P* Value^b^
Number of partners in past 3 months			
0 partners	36/204 (17.7)	34/190 (17.9)	
1 partner	135/204 (66.2)	119/190 (62.6)	
≥2 partners	33/204 (16.2)	37/190 (19.5)	0.61^c^
Unprotected sex at last sexd,e			
Spouse	5/55 (9.1)	55/98 (56.1)	<0.001*
Regular partner	3/59 (5.1)	33/68 (48.5)	<0.001*
Casual partner	10/65 (15.4)	15/25 (60.0)	<0.001*
Commercial partner	2/23 (8.7)	9/19 (47.4)	0.005*
Unprotected sex in past monthe,f			
0 acts	150/171 (87.7)	87/161 (54.0)	
1 acts	5/171 (2.9)	11/161 (6.8)	
2-5 acts	11/171 (6.4)	36/161 (22.4)	
6 or more acts	5/171 (2.9)	27/161 (16.8)	<0.001*^c^
Unsafe sex in past monthe,f			
0 HIV-negative or unknown-status partner	158/171 (92.4)	114/161 (70.8)	
1 HIV-negative or unknown-status partner	3/171 (1.8)	6/161 (3.7)	
2-5 HIV-negative or unknown-status partners	5/171 (3.5)	22/161 (13.7)	
≥6 HIV-negative or unknown-status partners	4/171 (2.3)	19/161 (11.8)	<0.001*^c^
Condom use self-efficacy			
Low self-efficacy (score 15-34)	2/204 (1.0)	0/190 (0)	
Moderate self-efficacy (score 35-54)	21/204 (10.3)	90/190 (47.4)	
High self-efficacy (score 55-75)	181/204 (88.7)	100/190 (52.6)	<0.001*^c^
HIV status disclosed to partnerd,e			
Spouse	52/55 (94.5)	89/98 (90.8)	0.41
Regular partner(s)	5/59 (8.5)	10/68 (14.7)	0.28
Casual partner(s)	6/65 (9.2)	9/25 (36.0)	0.002*
Commercial partner(s)	5/23 (21.7)	6/19 (31.6)	0.47
Currently receiving antiretroviral treatment	70/204 (34.3)	24/189 (12.7)	<0.001*
Internalized stigma			
Low (score 16-40)	90/203 (44.3)	107/190 (56.3)	
Moderate (score 41-52)	113/203 (55.7)	81/190 (42.6)	
High (score 53-64)	0/203 (0.0)	2/190 (1.1)	0.034*^c^

* *P* values < 0.05 were considered statistically significant.

a The following variables were used in propensity score matching: age, gender, education, religion, and employment.

b Pearson’s chi-square test unless otherwise indicated.

c Chi-square test for trend.

d Multiple-response question.

e Respondents were asked to report on all their sexual partners in the past 3 months with a maximum of 6 partners.

f Only among sexually active participants.

PLHIV in the intervention group were less likely those in the control group to report risky sex behaviors.

**Knowledge of HIV transmission** was higher in the intervention group than in the control group (median score = 5 [IQR: 2-5] versus 4 [IQR: 1-5], respectively; *P*<0.001; data not shown). At endline, more participants in the intervention arm than in the control arm exhibited high **condom use self-efficacy** scores (88.7% versus 52.6%), and had higher median scores on the knowledge of HIV transmission index (score = 5 [IQR = 2-5] versus 4 [IQR = 1-5]; *P*<0.001; data not shown).

More participants in the intervention arm than the control arm were **receiving ART** by endline (34.3% versus 12.7%; OR = 1.62, 95% CI = 1.07-2.46). Interestingly, a higher proportion of participants in the control group had low internalized **stigma** scores at endline compared with those in the intervention group (44.3% versus 56.3%). At endline, more participants in the intervention group than in the control group reported less **concern about HIV transmission** due to ART availability (84.2% versus 42.1%; OR = 7.3, 95% CI = 4.3-12.5; data not shown). Finally, analysis of outcomes separately by gender showed that all changes were in the same direction and of similar magnitude in women and men, with no differences detected in effect by gender.

## DISCUSSION

CHWs delivered a personalized HIV risk-reduction intervention that was successful in reducing reported risky behaviors (for example, improved condom use resulting in fewer unprotected sex acts and less unsafe sex) in a cohort of PLHIV who knew their HIV status but were not accessing HIV treatment or care services. The intervention also increased HIV knowledge and ART uptake.

Several studies have evaluated HIV-prevention interventions, but nearly all have been conducted among PLHIV recruited from HIV care or prevention services.^2^^,^^4-9^ To our knowledge, this is the first study to be conducted among PLHIV who are not accessing HIV services, with more than 40% of the participants testing positive with HIV more than 24 months previously.

Clients with HIV were recruited from the community, and the intervention was delivered by CHWs. While this is in variance with recommendations for successful HIV-prevention interventions made in a meta-analysis by Crepaz et al.,^4^ our findings provide evidence that non-formal health care providers can deliver interventions in non-clinical settings. The studies in the Crepaz review were conducted in the United States and suggested that provision of interventions by health care providers in service settings were effective in those settings.^4^ Shortage of human resources is a recognized limitation of health programs in several African countries and task shifting to other cadres is often considered. In this study, CHWs successfully delivered the intervention without any evidence of breaching confidentiality. Peltzer et al. (2010)^27^ also reported successful outcomes (increases in HIV knowledge, behavioral intentions, and risk-reduction efficacy, and declines in number of sex partners and unprotected sex) among persons with HIV receiving a risk-reduction intervention delivered by lay counselors at HTC services in South Africa.

Of note, nearly three-quarters of the participants were women, who constitute 60% of all adults with HIV in Kenya and in sub-Saharan Africa more broadly.^28^ There are several possible reasons for higher recruitment of women than men. For example, women might have been easier to reach in their homes by CHWs and more responsive to being included in a study, while men might have been more mobile. Also, more women might know their HIV status than men, often through contacts with the health system, such as antenatal clinics. Specific efforts are needed to recruit men, as they often make up a much smaller proportion within studies, HIV services, and health programs more generally. Employing more male CHWs might be useful.

Interestingly, in addition to the decline in numbers of reported partners in the intervention group, we observed a marginally significant reduction in the reported number of partners in the control group. It is possible that limited behavior change occurred among participants in the control group through the more limited contact with CHWs and participation in the research study. Nevertheless, future interventions should specifically focus on partner number as a critical concern.

Although reported disclosure of HIV status to spouses increased, disclosure to regular, casual, or commercial sex partners did not change in either group. Several other papers also report high disclosure rates within the marital union^29-31^ and lower rates of disclosure among previous or current casual partners.^31^ Within these transient or infrequent relationships, people might perceive a lower overall level of responsibility. While future research should explore disclosure and its context with casual and paid partners, the intervention could be further strengthened by specifically emphasizing disclosure to non-regular partners.

We noted a shift to lower internalized stigma scores in the intervention group compared with the control group in the study sample. However, the proportion of participants with low stigma scores was higher in the control group compared with the intervention group in the PSM sample at endline. The intervention focused on HIV risk reduction and emphasized disclosure to partners and family members. It is possible that frequent contact with CHWs and their guidance to disclose might have contributed to increased self-perceived stigma among intervention participants, at least in the short-term. In the long-term, however, disclosure of HIV status to spouses or main partners is associated with greater acceptance and support from partners, which includes anxiety relief, increased sexual communication, increased care-seeking behavior including ART uptake, and motivation to plan for the future among PLHIV.^31^^,^^33-34^

About half the participants in both groups expressed condom use fatigue, or being tired of always using condoms. The proportion of participants expressing condom use fatigue did not change over time and did not translate into an increase in unprotected sex; nevertheless, the impact of this over the long-term warrants concern.

### Study Limitations

The results should be interpreted in light of study limitations. Critically, the study relies on self-reported sexual risk and condom use behaviors, which might be subject to social desirability and recall bias. We used a 3-month recall period and limited the number of partners that each participant could report on to a maximum of 6, with the aim of obtaining more reliable recall and limiting the influence of outliers in the sample. Reviews of validity and reliability of HIV research show that sexual behavior data are fairly consistent and self-reported data on sexual acts are reasonably congruent, especially for short recall periods.^35-36^

Furthermore, a sizeable proportion of participants reported condom use fatigue, which could be considered to support the veracity of self-reported condom use behaviors. However, participants in the intervention group did receive additional counseling and follow-up, which might heighten desirability bias and improve recall in this group. The study would have benefited from clinical indicators or biological markers for unprotected sex to validate the results.

Further research is needed to demonstrate behavior change, not just reported behaviors. Our study did have one behavioral indicator (ART uptake) that showed a positive result in favor of the intervention.

We recruited PLHIV through non-probability targeted sampling using CHWs to reach potential participants, which might have introduced selection bias. Although our sample was not randomly recruited, we were able to reach PLHIV in the community, who are otherwise not accessible. Individual-level randomization would have controlled for the differences between the 2 groups at baseline. However, to remove the chance of intervention diffusion into the control group, we randomly assigned study sites to the 2 arms. Using PSM analysis, we were able to account for some differences, and findings were supported by results obtained from analysis over time in the entire study sample.

Some limitations of PSM warrant mention. The potential remains for unmeasured confounding and the smaller sample size resulting from this method limits the ability to detect smaller differences between the study groups.

The study tools had some limitations as well. The HIV knowledge index did not include an item on the awareness of HIV risk through sharing of needles/syringes among IDUs or unprotected sex with IDU partners. Recent studies have documented the presence of a sizeable IDU population in Mombasa, and future studies should include this information.^37-38^

Data were also not collected on some important variables such as CD4 cell count and access to health services. These variables could thus not be included in the PSM analysis. It is possible that the differences in ART uptake noted between the intervention and control site might have been due, in some part, to differences in baseline levels of CD4 cell count between participants in these areas.

Finally, given the specificities of the study location and the non-randomized study design, the findings of this research might not be generalizable to other settings. Moreover, our study sample was recruited from among PLHIV who know their HIV status, and our findings might not be applicable to PLHIV who have not been tested.

Community-based delivery of positive prevention interventions shows promise for large-scale replication in resource-limited settings.

## CONCLUSION

In conclusion, the community-based positive prevention intervention effectively reduced reported risky sexual behavior and increased ART uptake among PLHIV who knew their HIV status. As the intervention was delivered by non-formal health care providers from the community using a national CHW training package, we consider it suitable for large-scale replication in similar resource-limited settings. Strategies to reach more PLHIV in the community, especially men who are not accessing services, need further research, as do the effects of interventions that target structural and other forms of vulnerability, in addition to the behavioral interventions applied here. Finally, the long-term effects and sustainability of this intervention warrant further assessment, perhaps within a longitudinal study.
